# ASME B89.4.19 Performance Evaluation Tests and Geometric Misalignments in Laser Trackers

**DOI:** 10.6028/jres.114.003

**Published:** 2009-02-01

**Authors:** B. Muralikrishnan, D. Sawyer, C. Blackburn, S. Phillips, B. Borchardt, W. T. Estler

**Affiliations:** Precision Engineering Division, National Institute of Standards and Technology, Gaithersburg, MD 20899

**Keywords:** ASME B89.4.19, geometric errors, laser tracker, misalignments, performance evaluation, sensitivity analysis

## Abstract

Small and unintended offsets, tilts, and eccentricity of the mechanical and optical components in laser trackers introduce systematic errors in the measured spherical coordinates (angles and range readings) and possibly in the calculated lengths of reference artifacts. It is desirable that the tests described in the ASME B89.4.19 Standard [1] be sensitive to these geometric misalignments so that any resulting systematic errors are identified during performance evaluation. In this paper, we present some analysis, using error models and numerical simulation, of the sensitivity of the length measurement system tests and two-face system tests in the B89.4.19 Standard to misalignments in laser trackers. We highlight key attributes of the testing strategy adopted in the Standard and propose new length measurement system tests that demonstrate improved sensitivity to some misalignments. Experimental results with a tracker that is not properly error corrected for the effects of the misalignments validate claims regarding the proposed new length tests.

## 1. Introduction

A spherical coordinate measurement instrument such as a laser tracker is a mechanical assembly of several components that may contain misalignments within its construction. These geometric offsets, tilts and eccentricities introduce systematic errors in the measured spherical coordinates (angles and range readings) and possibly in the calculated lengths of reference artifacts. It is general practice to compensate for these errors through software error models [2–4] in a manner somewhat similar to that performed for Cartesian coordinate measuring machines (CMMs).

Early designs for laser trackers used a mirror mounted on a two-axis gimbal mechanism to steer the beam to the target. Several sources of geometric misalignments for this configuration were identified by Loser and Kyle [5], and an error model was developed and published. However, the presence of a geometric error model within the system does not necessarily imply the absence of systematic errors. Incorrect compensation or misalignments after compensation are possible and have to be identified during performance evaluation.

The recently introduced ASME B89.4.19 Standard proposes a common set of ranging tests, length measurement system tests and two-face system tests, which can be performed by manufacturers and users to assess the performance of their instrument. It is desirable that the tests described in the B89.4.19 Standard be sensitive to the different potential error sources, including errors resulting from geometric misalignments within the tracker. This paper addresses the issue of the sensitivity of the tests described in the B89.4.19 Standard to geometric misalignments within a tracker.

## 2. Approach

The corrected (or true) range (*Rc*) and angles (*Hc*, *Vc*) of any coordinate in space are functions of several misalignment parameters within the construction of the tracker and also of the measured coordinate values at that location (*Rm*, *Hm*, *Vm*). The corrections Δ*Rm*, Δ*Hm* and Δ*Vm* in *Rm*, *Hm* and *Vm* respectively may be expressed as

$$\begin{array}{l} Rc-Rm=\Delta Rm=f_{r}(Rm,Hm,Vm,x_{1},x_{2},\ldots ,x_{n})\\ Hc-Hm=\Delta Hm=f_{h}(Rm,Hm,Vm,x_{1},x_{2},\ldots ,x_{n})\\ Vc-Vm=\Delta Vm=f_{v}(Rm,Hm,Vm,x_{1},x_{2},\ldots ,x_{n}) \end{array}$$

where *x_i_* (*i* = 1 to *n*) are *n* misalignment parameters. The above constitutes an error model for a laser tracker. Loser and Kyle’s model containing 15 parameters is one such error model applicable to laser trackers with a beam steering mirror. Since the design of such trackers in the 1980s, trackers with other mechanical configurations have emerged. These trackers have different error models, but manufacturers have been reluctant to publish them. We have extended Loser and Kyle’s model to two other common configurations also.

The development of an error model for a laser tracker immediately suggests a numerical approach to sensitivity analysis, where misalignment parameters are perturbed while determining the impact on each of the performance evaluation tests, which are also numerically simulated. We have performed such analysis for three different mechanical constructions of trackers: a) a tracker with a beam steering mirror for which the Loser and Kyle model is applicable, b) a tracker with the laser source in the rotating head and c) a scanner with source mounted on the transit axis with a rotating prism mirror that steers the beam to the target.

In this paper, we describe our analysis for only the tracker with the laser source in the rotating head of the instrument (or other constructions such as those with a fiber coupled laser that may also be modeled in the same manner). Such a tracker can be imagined to have a theodolite-type construction where the telescope in the rotating head is replaced by a laser source. The simple geometric design enhances clarity in our presentation; the method is applicable to the other designs as well.

The organization of the remainder of the paper is as follows. We briefly describe the ASME B89.4.19 tests in Sec. 3. We describe the coordinate system adopted in Sec. 4. In Sec. 5, we detail geometric misalignment parameters relevant to the chosen configuration of the tracker, and the different two-face and length measurement system tests that are sensitive to those terms. We present an error model for the tracker in Sec. 6. The sensitivity of any geometric misalignment parameter to each of the tests described in the Standard is compiled in the form of a matrix and presented in Sec. 7. A summary of our observations from the analysis in Sec. 5 and the sensitivity matrix in Sec. 7 is presented in Sec. 8. We propose a new set of length measurement system tests that demonstrate improved sensitivity in Sec. 9. In Sec. 10, we discuss experimental results that validate some of our proposed new length tests. Our conclusions are summarized in Sec. 11.

## 3. The ASME B89.4.19 Standard

The B89.4.19 Standard describes three kinds of tests to be performed on laser trackers—ranging tests, length measurement system tests and two-face system tests. The ranging tests assess the distance measuring capability of the tracker along a purely radial direction. We do not consider ranging tests in this paper as these are not diagnostic of geometric misalignments in a tracker.

Length measurement system tests are performed to assess the tracker’s ability to measure different lengths within the work volume. Because these tests exercise the kinematic links in the tracker, they are sensitive to most of the tracker’s geometric misalignments. The Standard requires length measurement system tests to be performed in 33 predetermined and two user-defined positions.

There are a number of geometric misalignments that produce angle errors that reverse in sign between a front face and back face measurement of the tracker. Two-face tests are therefore excellent diagnostics of these geometric misalignments. The Standard requires that two-face errors be measured at 36 predetermined positions.

## 4. Coordinate System Definition

**Fig. 1:**
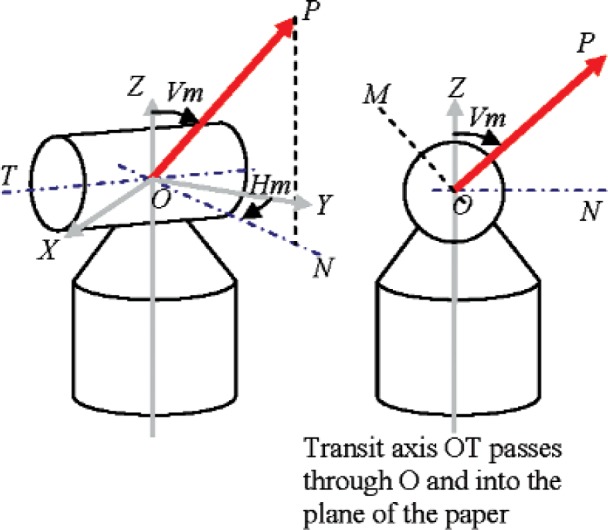
Coordinate system definition.

In Fig. 1, the *X*, *Y*, and *Z* axes form a mutually orthogonal fixed Cartesian system with its origin at *O*. We define two axes—the transit axis *OT* and its normal in the *XY* plane *ON*—attached to the instrument head that can rotate about the standing axis *OZ*. The target is located at *P*. In addition, we also define an axis *OM* attached to the instrument head. *OM* is orthogonal to the transit *OT* and the beam path *OP*. Horizontal angles are measured from the *Y* axis to *ON*. Vertical angles are measured from the *Z* axis to *OP*. It should be noted that in an ideal case, transit axis *OT* intersects, and is perpendicular to the standing *OZ*. The beam emerges from *O* and its path to the target *OP* is perpendicular to the transit axis *OT*. In reality, any or all of these conditions may be violated, resulting in systematic errors in the measured range and angles.

## 5. Geometric Misalignment Parameters and Sensitivity to B89.4.19 Tests

In this section, we discuss the effect of several geometric misalignment parameters in a theodolite-type tracker on the measured range and angles. We also discuss two-face and length measurement system tests that are sensitive to each of the misalignment parameters.

### 5.1 Beam Offset (*x*_1_)

#### Description

**Fig. 2:**
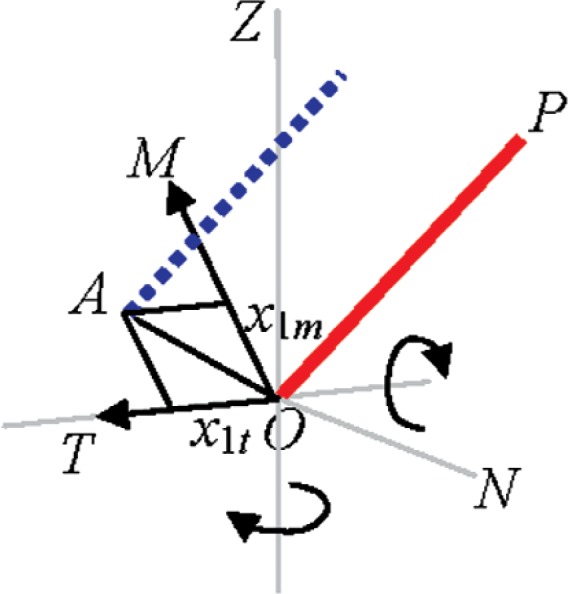
Beam offset.

The beam originating from the source (at *O*) may be displaced from its ideal position by a constant offset (*OA* in Fig. 2) to emerge from *A*, a misalignment parameter referred to as beam offset. The offset can be resolved into components along *M* and *T* axes (*x*_1_*_m_* and *x*_1_*_t_*) because the beam originates in the rotating head traveling parallel to *OP* and perpendicular to the *MOT* plane in the *MTP* coordinate system. The offset component along the transit (*x*_1_*_t_*) produces an error in the measured horizontal angle. The correction for the beam offset is given by $\Delta Hm=\frac{x_{1t}}{Rm\sin(Vm)}$ (positive *x*_1_*_t_* is as shown in Fig. 2; therefore the measured horizontal angle is smaller than the true angle, which produces a positive correction). The component along its normal (*x*_1_*_m_*) produces an error in the measured vertical angle, and its correction is given by $\Delta Vm=\frac{-x_{1m}}{Rm}$.

#### Two-face system tests

The corrections for the measured horizontal and vertical angles of a target placed distance *Rm* away from the front face of the tracker are given in the preceding sub-section. These corrections reverse in sign in the back face of the tracker. The apparent distance *E* in a two-face system test is therefore given by *E* = 2Δ*HmRm*sin(*Vm*) = 2x_1_*_t_* for an offset along the transit axis and *E* = 2Δ*VmRm* = 2*x*_1_*_m_* for an offset along *OM*. Every two-face system test described in the Standard is therefore sensitive to both beam offset parameters by the same sensitivity factor of 2.

#### Length measurement system tests

Systematic errors in measured range and angles lead to an error in the determination of the coordinates of each end of the reference length. This however does not necessarily imply an error in the calculated length between the two ends, because the error vectors at the two ends may simply result in translation and/or rotation of the length without a change in its magnitude (the error vector is the vector between true coordinate and measured coordinate). Sensitivity to length measurement test is achieved primarily if the error vectors at the two ends produce components along the length with a non-zero sum. Components perpendicular to the length generally do not produce a significant change in length for the test to be sensitive to the geometric misalignment under consideration.

Any symmetrically placed reference length (such as the horizontal, vertical or diagonal length tests in the Standard) is not sensitive to beam offset, because they only serve to translate and rotate the length. The default position for the first user-defined test (asymmetrical vertical length test) is sensitive to beam offset along the *M* axis because the asymmetrical positioning of the reference length creates unequal error components at the two ends of the reference length, which do not completely cancel each other. We discuss such asymmetrical positioning in Sec. 9.

### 5.2 Transit Offset (*x*_2_)

#### Description

**Fig. 3:**
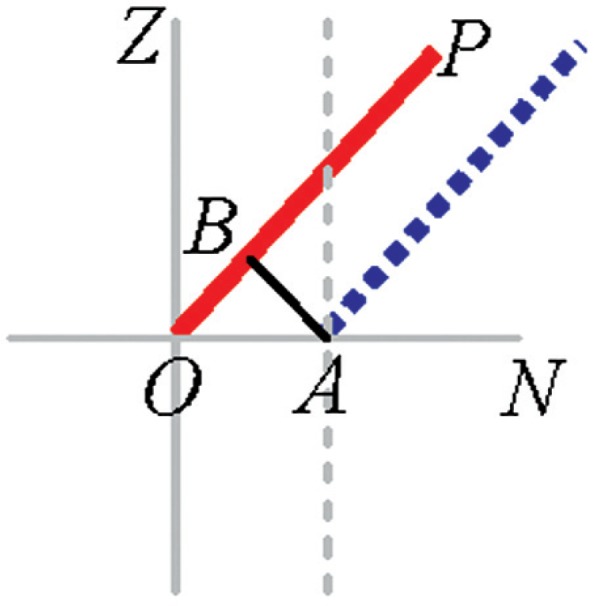
Transit offset.

The transit axis may be offset from its ideal location and therefore not intersect the standing axis. This offset is referred to as the transit offset (*OA* in Fig. 3). The transit offset vector of consequence is along the *N* axis. The offset along *Z* is simply a translation of the coordinate system. Transit offset produces an error in the measured range (*OB*). The correction in the range is given by Δ*Rm* = *x*_2_ sin(*Vm*). Additionally, the transit offset produces an error in the measured vertical angle (vertical angle error = *AB/AP*). The correction in vertical angle is given by $\Delta Vm=\frac{x_{2}\cos(Vm)}{Rm}$. For a positive offset as shown in Fig. 3, the measured range and vertical angle are smaller than the true values, hence a positive correction.

#### Two-face system tests

The transit offset is sensitive to two-face measurements, because the error in the measured vertical angle changes in sign between the two faces. The two-face error *E* is given by *E* = 2*Rm*Δ*Vm* = 2 *x*_2_ cos(*Vm*). The test is not sensitive in the horizontal plane (*Vm* = 90°, cos(*Vm*) =0). Sensitivity is higher near the pole and also near the floor but decreases farther away from the tracker (because the target continues to remain at the same height and therefore *Vm* increases). Note that theoretically, range measurements are smaller in one face and larger in the other and therefore might seem to also contribute to a two-face error. In practice, range measurements are not recorded in the back face (for interferometric systems, a break in the beam precludes such measurements) and therefore range related effects are not diagnosed in these tests.

#### Length measurement system tests

A ranging test will not detect transit offset because point-to-point distances are unaffected along the radial direction. A horizontal length test, however, captures some of this term. Higher sensitivity is obtained when the reference length is placed as close as possible to the tracker. In fact, if the tracker is placed between the targets and in line with them, we achieve a maximum sensitivity of 2. More generally, the sensitivity to horizontal length tests as described in the B89.4.19 Standard can be given by $\frac{L}{\sqrt{\frac{L^{2}}{4}+D^{2}}}$, where *L* is the value of the reference length (2.3 m) and *D* is the distance between the reference length and the tracker.

### 5.3 Vertical Index Offset (*x*_3_)

#### Description

A shift in the zero of the vertical angle encoder from the pole results in a constant error in the measured vertical angle, a misalignment parameter referred to as vertical index offset.

#### Two-face system tests

The vertical angle error changes in sign between front face and back face measurements. The apparent distance between front face and back face measurements of the target scales with distance and is given by *E* = 2*Rmx*_3_. There is increased sensitivity farther away from the tracker.

#### Length measurement system tests

Vertical index offset results in a constant vertical angle error in every measured point. None of the length measurement system tests described in the Standard will capture this error because it manifests primarily as a coordinate rotation for the positions and orientations of the lengths described in the Standard. In Sec. 9, we describe a length test that is sensitive to this parameter.

### 5.4 Beam Tilt (*x*_4_)

#### Description

**Fig. 4:**
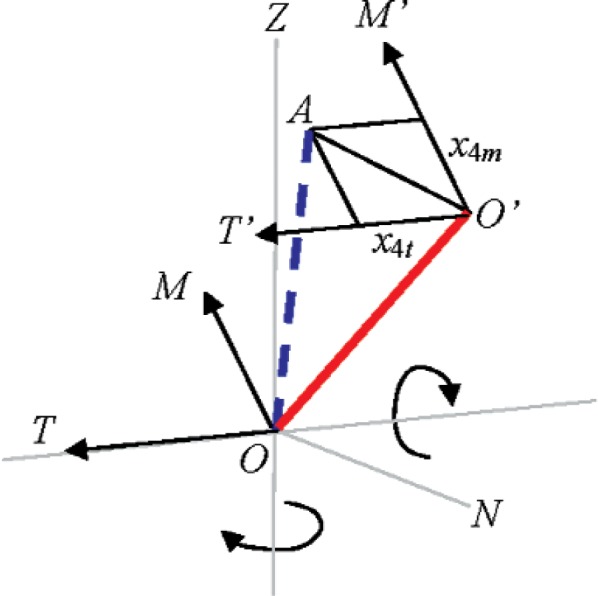
Beam tilt.

The beam emerging from the source may be tilted (not normal to the transit axis) from its ideal path. This is a misalignment referred to as beam tilt. Although shown in Fig. 4 as an offset, we define *x*_4_ as the offset per unit length (along the beam path to target), and it therefore may be expressed in units of angle (small angle approximations will be valid). Beam tilt may be resolved into components in a similar manner to beam offset (*x*_4_*m* and *x*_4_*t*). However, the component along *M* is indistinguishable from the vertical index offset and is therefore not considered. The component along *T* is equivalent to collimation error in theodolites. The correction in the measured horizontal angle is given by $\Delta Hm=\frac{x_{4t}}{\sin(Vm)}$ [6] For positive tilt as shown in Fig. 4, the measured horizontal angle is smaller, hence a positive correction.

#### Two-face system tests

The apparent distance *E* in a two-face system test is given by *E* = 2*Rm* sin (*Vm*) Δ*Hm* = 2*Rmx*_4_*_t_*, and scales with distance as expected. There is increased sensitivity farther away from the tracker.

#### Length measurement system tests

None of the length measurement system tests described in the Standard is sensitive to this parameter. A reference length placed such that both ends are at the same *Z* height (horizontal length test) will have identical errors at the two ends and therefore be insensitive to this parameter. An asymmetric vertical length test is also insensitive because the horizontal angle errors, although different at the two ends, are directed normal to the length; errors directed axially (along the length) will be most sensitive. A diagonal test where the two ends are at different *Z* heights is desirable. However, symmetrically placed diagonals such as in the B89.4.19 Standard are not sensitive, because the errors are identical at the two ends (sin(*Vm*) = sin(π−*Vm*)). Asymmetric diagonals that are sensitive to this parameter are described in Sec. 9.

### 5.5 Transit Tilt (*x*_5_)

#### Description

The transit axis, although intersecting the standing axis, may be tilted (not at a right angle) relative to the standing axis. This lack of squareness between the axes is referred to as transit tilt and is also a common theodolite error source. Again, as in beam tilt, the parameter is a ratio of offset per unit length. It is expressed in radians using the small angle approximation. Lack of squareness between the two axes produces an error in the measured horizontal angle. The correction is given by $\Delta Hm=\frac{x_{5}}{\tan(Vm)}$ [6].

#### Two-face system tests

The transit tilt produces an error in the horizontal angle that is sensitive to two-face measurements. The apparent distance in a two-face measurement is given by $E=2Rm\sin(Vm)\Delta Hm=2Rm\sin(Vm)\frac{x_{5}}{\tan(Vm)}=2Rmx_{5}\cos(Vm)$. For the two-face system tests described in the Standard, there is no sensitivity when a test is performed at the tracker height (*Vm* = 90°, cos (*Vm*) = 0). For all other positions as described in the Standard, the sensitivity is a constant, because *Rm* cos (*Vm*), which is the projection of the beam vector along the *Z* direction, is a constant.

#### Length measurement system tests

The transit tilt is analogous to squareness error in an *XY* stage. The diagonal length tests described in the Standard are sensitive to this parameter. The left and right diagonal tests provide errors that are equal in magnitude but opposite in sign as a consequence of transit tilt. The sensitivity does not change with distance from the tracker because although the range to the targets increases, the vertical angles also change (the reference length is same).

### 5.6 Encoder Eccentricity (*x*_6_ and *x*_7_)

#### Description

**Fig. 5:**
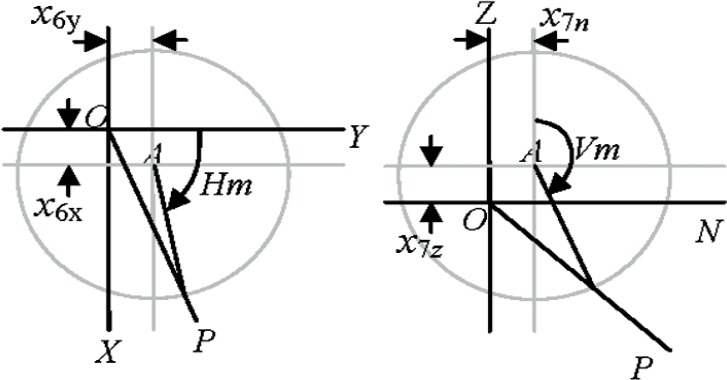
Horizontal and vertical angle encoder eccentricity.

The horizontal and vertical angle encoders may be eccentrically mounted, parameters we refer to as horizontal and vertical angle encoder eccentricity. We describe the terms as a ratio of the actual eccentricity to the radius of the encoder so that *x*_6_ and *x*_7_ are dimensionless quantities. The horizontal angle encoder is fixed to the base and its eccentricity (*OA*) can therefore be resolved into *X* and *Y* components (*x*_6_ *_x_* and *x*_6_ *_y_*). The vertical angle encoder is fixed to the moving transit and, consequently, the physical encoder eccentricity can be resolved into *N* and *Z* components (*x*_7_ *_n_* and *x*_7_ *_z_*). For the coordinate system in Fig. 5, it can be shown that the correction in the measured horizontal angle due to the horizontal angle encoder eccentricity is Δ*Hm* = *x*_6_ *_x_* cos (*Hm*) − *x*_6_ *_y_* sin (*Hm*), and the correction in the measured vertical angle due to the vertical angle encoder eccentricity is Δ*Vm* = *x*_7_ *_n_* cos(*Vm*) − *x*_7_ *_z_* sin (*Vm*).

#### Two-face system tests

The apparent distance measured between the two faces of the tracker in the case of horizontal angle encoder eccentricity is given by 2(*Rm* sin (*Vm*)) *x*_6_ *_x_* cos (*Hm*) for eccentricity along *X* and 2(*Rm* sin (*Vm*)) *x*_6_ *_y_* sin (*Hm*) for eccentricity along *Y*. The error increases farther away from the tracker and may drop to zero depending on the azimuthal angle. The apparent distance in the case of vertical angle encoder eccentricity along *N* is also given by a similar expression, 2*Rmx*_7_ *_n_* cos (*Vm*). Note, however, that vertical angle encoder eccentricity along *Z* is not sensitive to two-face measurements. It is of interest to note that two-face system tests, if applicable, are sensitive to both the azimuth and the distance of the target from the tracker.

#### Length measurement system tests

A horizontal length test as described in the B89.4.19 Standard positioned parallel to the *X* axis is sensitive to the horizontal angle encoder eccentricity along the *Y* axis (*x*_6_ *_y_*) because the horizontal angle subtended by the length at the origin is different from the true angle. Similarly, a horizontal length positioned parallel to the *Y* axis is sensitive to the horizontal angle encoder eccentricity along the *X* axis. A vertical length test as described in the Standard is sensitive to vertical angle encoder eccentricity along *N* for the above reason. None of the pre-determined positions for length measurement system tests in the Standard is sensitive to vertical angle encoder eccentricity along *Z*, because the symmetrical positioning produces error vectors that only translate/rotate the length. The asymmetrical positioning of the vertical length, such as in the user-defined default position 1, is somewhat sensitive. We discuss this in Sec. 9.

### 5.7 Bird Bath Error (*x*_8_)

#### Description

The bird bath error is a constant error in the range caused by incorrect calibration of the distance to the fixed reference point (bird bath) within the tracker.

#### Two-face system tests

This term is not sensitive to two-face system tests.

#### Length measurement system tests

Because range measurements are either smaller or larger than the true value, all length measurement system tests produce length results that are either smaller or larger than the true value. The sensitivity is higher when the reference length is closer to the tracker because a larger component of the error vector is along the length. The highest sensitivity (twice the bird bath error) is obtained when the tracker is placed in line between the targets, as in the case of transit offset.

### 5.8 Scale Errors in the Encoder (*x* 9 and *x* 10)

#### Description

If scale errors are present, lower order harmonics may be the largest contributors to errors in measured angles. The correction in the measured angles due to the presence of *m*^th^ order harmonic errors in the scales may be expressed as Δ*Hm* = *x*_9_ *_a_* sin(*m.Hm*) + *x*_9_ *_b_* cos(*m.Hm*) for the horizontal angle encoder and Δ*Vm* = *x*_10_ *_a_* sin(*m.Vm*) + *x*_10_ *_b_* cos(*m.Vm*) for the vertical angle encoder. The first order (*m* = 1) is indistinguishable from encoder eccentricity. We only consider second order harmonics in the discussion below and in the error model in the next section.

#### Two-face system tests

Two-face system tests are sensitive to odd order harmonic errors while they are not sensitive to even order harmonic errors. Therefore, length measurement system tests are important to identify second and higher order even harmonics.

#### Length measurement system tests

Horizontal length tests as described in the Standard are sensitive to one component of the second order harmonic error, *x*_9_ *_a_* sin(2*Hm*). Horizontal length tests performed 45° away in azimuth will be sensitive to the other component, *x*_9_ *_b_* cos(2*Hm*). The Standard however does not specify length measurement system tests at any azimuthal angle other than 0°, 90°, 180° and 270°. As an alternative, an asymmetrical length test as described in Sec. 9 may be performed as a diagnostic for this parameter. Vertical length tests described in the Standard are sensitive to one component of the second order harmonic error, *x*_10_ *_a_* sin(*2Vm*). The user defined default position 1, which is the asymmetrical vertical length test described in Sec. 9, is sensitive to the other component, *x*_10_ *_b_* cos(*2Vm*).

## 6. Error Model

The above described terms can be combined into the following error model, which is an adaptation of the Loser and Kyle model [5] for trackers with a beam steering mirror (model applicable for front face measurements only):

$$\begin{array}{l} Rc=Rm+x_{2}\sin(Vm)+x_{8}\\ Hc=Hm+\frac{x_{1t}}{Rm.\sin(Vm)}+\frac{x_{4t}}{\sin(Vm)}+\frac{x_{5}}{\tan(Vm)}+x_{6x}\cos(Hm)-x_{6y}\sin(Hm)+x_{9a}\sin(2Hm)+x_{9b}\cos(2Hm)\\ Vc=Vm-\frac{x_{1m}}{Rm}+\frac{x_{2}\cos(Vm)}{Rm}+x_{3}+x_{7n}\cos(Vm)-x_{7z}\sin(Vm)+x_{10a}\sin(2Vm)+x_{10b}\cos(2Vm) \end{array}$$

## 7. Sensitivity Matrices

The preceding analysis and error model suggest a numerical approach to sensitivity analysis where each misalignment parameter is individually perturbed to assess its impact on all performance evaluation tests, which are also numerically simulated. We performed this analysis, and the results are provided in Tables 1 and 2. The information is presented in matrix form, so the relationship between the sensitivity for each misalignment parameter and the performance tests is easily obtained.

Care must be taken in interpreting the sensitivity values. For geometric offsets, sensitivity represents error in micrometers for 1 μm of offset. For tilt terms, sensitivity represents error in micrometers for 1 μrad of tilt. For eccentricity terms, sensitivity represents error in micrometers for one non-dimensional unit of eccentricity (eccentricity itself is a ratio of the offset in micrometers to radius of the encoder, also in micro-meters).

Columns in the table correspond to the misalignment parameters while rows correspond to tests described in the Standard. Thus, the entry in row 3 (corresponding to Test # 3), column 3 (corresponding to *x*_2_) implies a sensitivity of −0.7 for the horizontal length test (3 m, 90° azimuth) to transit offset parameter (see note below tables to interpret rows).

In the next section, we make several observations based on the preceding analysis and the sensitivity matrices shown. It should be noted that the sensitivity matrices are different for different tracker constructions (different error models) and we therefore caution against extensive generalization from the one reported instance here. The observations in the next section come from an analysis of the three commonly found tracker configurations, which we have modeled.

## 8. Discussion

### Two-face system tests

A large number of geometric misalignment parameters are sensitive to two-face measurements. These tests are easy to perform and require no calibrated reference artifacts. Two-face system tests are therefore a critical component in a performance evaluation of laser trackers.

Some geometric misalignment parameters are equally sensitive to two-face measurements at all distances from the tracker while others show increasing sensitivity farther away from the tracker. Some parameters are insensitive to the azimuth or elevation, while others are sensitive. The tests described in the B89.4.19 Standard capture these influence parameters effectively by requiring the target be positioned at different distances from the tracker, and at different horizontal and vertical angles.

It should be pointed out that the sensitivity values were generated by considering the influence of only one misalignment parameter at a time, and each of unit value. The error in any two-face system test in the presence of two or more misalignment parameters at a time (each of unit value) is not necessarily given by the sum of the individual sensitivities for two reasons.

First, the distance error is determined as the root sum square of the errors in two orthogonal directions; one component is due to the horizontal angle error and another due to vertical angle error. Therefore, summation without regard to direction will produce erroneous results. Second, the two-face error, which is defined as the magnitude of the vector joining the coordinate determined from the front face measurement to that determined from the back face measurement, disregards the sign of the vector. Therefore, two or more parameters that produce non-zero error individually may in combination result in cancellation of the error.

The convolution of the angle errors into a distance error may result in unification of the reporting method with length measurement system tests within the Standard. But the above observations motivate us to suggest that angle errors be recorded in addition to distance errors in two-face system tests as they have significant diagnostic value.

For the tracker mechanical configuration we discussed in this paper, there are only a few misalignment parameters that are insensitive to two-face system tests (vertical angle encoder eccentricity along *Z*, bird bath, and second order scale error). For other configurations such as those with rotating mirrors, there are many terms that are not sensitive to two-face system tests (see [5]). It is therefore desirable to have a set of length measurement system tests that are also sensitive to most or all of the geometric misalignment parameters in a tracker.

### Length measurement system tests

From the discussion in the previous section and the sensitivity matrix in Table 1, it is clear that certain misalignment parameters are detected by some of the length measurement system tests described in the Standard (three out of four encoder eccentricity parameters, transit tilt, transit offset, and some second order scale error components). There however appears to be reduced or no sensitivity to other geometric misalignment parameters (beam offsets, beam tilt, vertical index offset, vertical encoder eccentricity along *Z*). Small changes in the position and orientation of the reference lengths greatly improve sensitivity as shown in the next section.

Another observation may be made from Table 1. From a geometric error modeling perspective alone, it appears that there is no value in performing vertical length tests at different orientations of the tracker (0°, 90°, 180° and 270°) because the vertical length tests are never a function of the azimuth. Further, the value in performing vertical length tests at different distances from the tracker is also not apparent.

Finally, we note that unlike in the case of two-face errors, the error in any length measurement system test in the presence of multiple geometric misalignment parameters (each of unit value) is indeed given by the sum of the individual sensitivities. This is true because the sensitivity to any given geometric misalignment parameter is determined only from the component of the error vector along the length (perpendicular components are not sensitive), and these scalar components can be summed algebraically.

## 9. Proposed New Length Measurement System Tests

We propose in this section some new length measurement system tests that demonstrate improved sensitivity to some of the geometric misalignment parameters previously undetected. It should be pointed out that the tests described here may be sensitive to multiple misalignment parameters, but we highlight only one or two misalignment parameters for each test for purposes of illustration. Further, we re-emphasize that sensitivity is a function of the mechanical design employed, and the description below pertains only to the theodolite-type tracker we have considered in this paper.

### 9.1 Asymmetrical Horizontal Length Test (Rows A1, A2 in Table 1)

We mentioned earlier that beam offset along the transit axis is insensitive to symmetrically placed reference lengths because the error vectors at the two ends only serve to translate and rotate the length, not change its magnitude. In fact, we noted that there is no length measurement system test in the B89.4.19 Standard that is sensitive to this term.

**Fig. 6:**
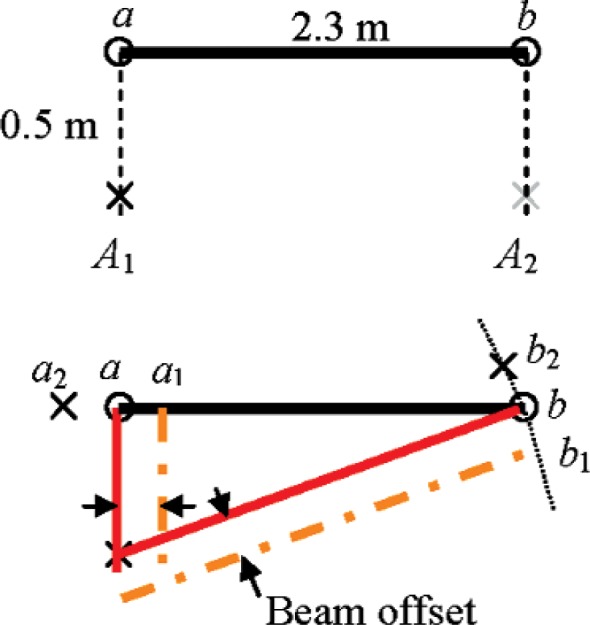
Asymmetrical horizontal length test (top view shown).

A small modification in the positioning of the reference length in the horizontal length test addresses this issue. Instead of being placed at the center of the reference length, the tracker is placed near one end and as close as possible (0.5 m) to the length. Figure 6 shows a schematic of the setup with the tracker at *A*_1_ and the two ends of the reference length at *a* and *b*. Also shown in the figure is the offset beam pointing at *a*_1_ instead of at *a*. In rotating the tracker so that the beam points to *a*, the target appears to be at *a*_2_ in the tracker coordinate system. A similar effect occurs at target *b* also. This test is sensitive to beam offset, because the projections of the errors along the reference length are much larger at *a* than at *b*.

In addition to the position *A*_1_, we also define a mirror position *A*_2_ where the tracker can be placed and the length re-measured. The error at this location of the tracker is equal in magnitude but opposite in sign to that measured with the tracker at *A*_1_. Two quick measurements can therefore provide substantial diagnostic information.

This test, as mentioned earlier, is sensitive to several geometric misalignment parameters. Of these, it is worth mentioning that this test captures a component of the second order scale error, *x*_9_*_b_* cos (2*Hm*), not detected by other tests.

### 9.2 Asymmetrical Vertical Length Test (Rows B1, B2 in Table 1)

An argument similar to the above may be made for beam offset along the *M* axis where an asymmetrical vertical length test is sensitive, while other tests are not. As mentioned earlier, such a test is described in the Standard as the user defined default position 1. We suggest the tracker be placed as close as possible to the 2.3 m reference length and level with the lower target (*Vm* = 90°). Our experiments suggest a 1 m distance is practical; hence the lower sensitivity in Table 1 in comparison with the asymmetrical horizontal length test for which we use a 0.5 m distance. Again, there is a mirror position (where the tracker is placed at the high point so that *Vm* = 90° for the target at the top) for this test where the error reverses in sign, although in practice it may be more difficult to mount the tracker at this height.

### 9.3 Asymmetrical Diagonal Test (Rows C1, C2 in Table 1)

**Fig. 7:**
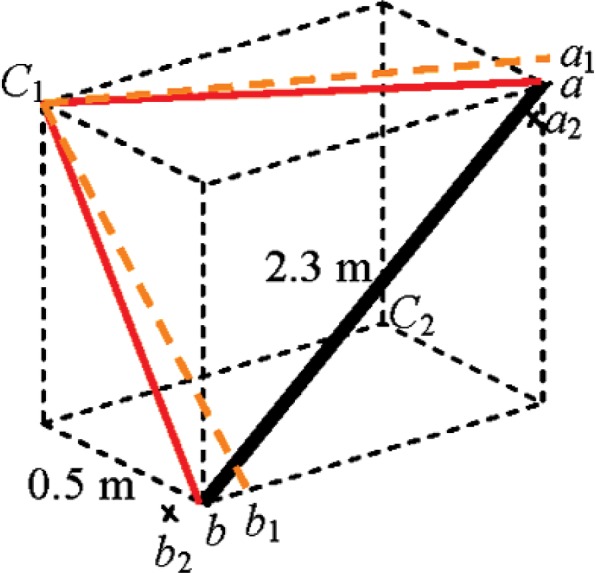
Asymmetrical diagonal length test.

As mentioned earlier the beam tilt parameter (collimation error in theodolites) is not captured by any of the B89.4.19 length measurement system tests. We suggested that an asymmetrical diagonal test is somewhat sensitive to this term. Fig. 7 shows a schematic of such a test. In Fig. 7, with the tracker at *C*_1_ and target at *a*, let the beam tilt result in the beam pointing at *a*_1_. The effect of this error is that the target appears to be at *a*_2_ in the tracker coordinate system. There is a similar effect for the target at *b* also. However, the error vectors *a*−*a*_2_ and *b*−*b*_2_ have different directions and magnitudes (the horizontal angle errors are not the same at *a* and *b* because of the different vertical angles; recall that the correction $\Delta Hm=\frac{x_{4t}}{\sin(Vm)}$). Each error term contributes a different component along the length. The residual provides the sensitivity. Again, there exists a mirror position *C*_2_ where the error reverses in sign.

### 9.4 Horizontal Length Above the Tracker (Rows D1, D2 in Table 1)

**Fig. 8:**
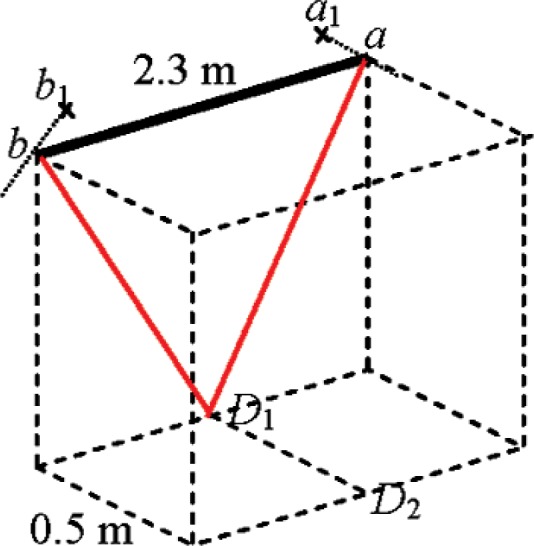
Horizontal length above tracker.

The horizontal length test above the tracker (tracker at *D*_1_ in Fig. 8) is particularly sensitive to two misalignment parameters generally not sensitive to the length measurement system tests described in the Standard -vertical encoder eccentricity along *Z* and vertical index offset.

With the tracker at *D*_1_ and target at *a*, assume either of the two misalignment parameters mentioned above causes the point to appear at *a*_1_. Rotation about the standing axis results in the same effect with target at *b* appearing to be at *b*_1_. In case of vertical index offset, there is either a dead space in the encoder near the pole or an overlap of angle resulting in a length error. The components of the error vectors *a*−*a*_1_ and *b*−*b*_1_ along the reference lengths sum to make this test sensitive to these parameters. A practical realization for this test, if the above were not feasible, would be to place the tracker as close as possible to the horizontal reference length (say at *D*_2_).

### 9.5 Tracker in Line and in Between Targets (Row E in Table 1)

The standard requires the measurement of a horizontal length with the tracker as close as possible to the reference length. This test is particularly sensitive to transit offset and the bird bath error if the tracker is placed in between the target nests and in line with the targets.

## 10. Experimental Validation

**Fig. 9:**
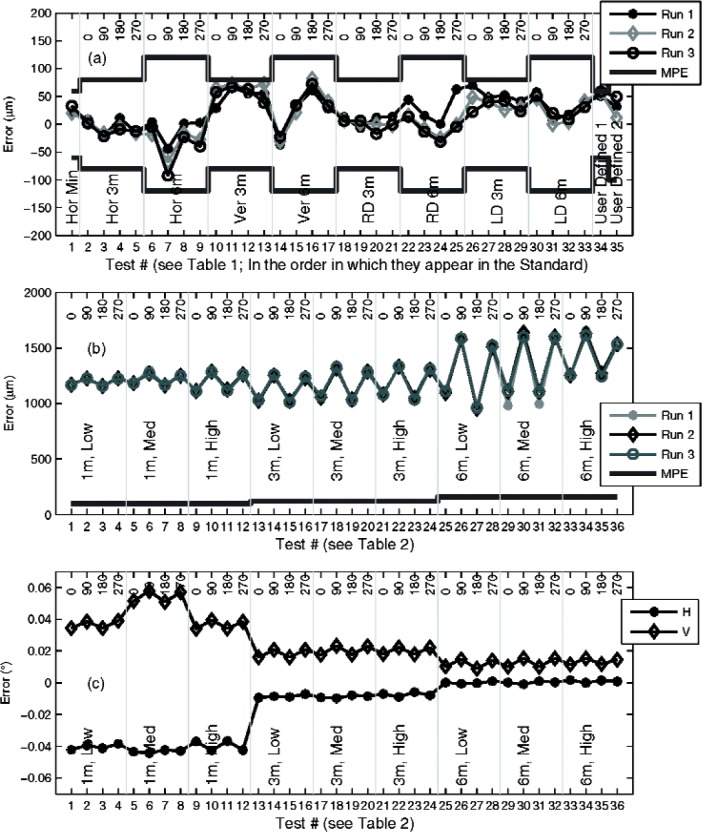
(a) Length measurement system test results (b) Two-face system test results (c) Angle errors in two-face data.

The B89.4.19 length measurement system test results and two-face system test results for a tracker are presented in Fig. 9(a) and Fig. 9(b). Notice that the tracker passed the length measurement system test portion of the B89.4.19 Standard but showed large errors in the two-face system tests. We extracted the raw horizontal and vertical angle data for the two-face system tests; they are shown in Fig. 9(c). Note that the angular errors scale inversely with distance.

Based on our models, the likely cause for this behavior is an offset in the beam as it emerges from the head (a constant offset implies reducing angular errors with increasing distance). The beam is likely offset along both the transit axis *OT* and along its normal *OM* (as defined in Fig. 1). From the measured angle errors and range values, we compute the actual beam offsets within the instrument head along the two directions to be 473 μm and 359 μm using a least-squares best fit. The precise offset values are not relevant; approximate values are useful in understanding the impact on length measurements made in the working volume of the tracker.

We performed the asymmetrical horizontal and the asymmetrical vertical length tests as described in Sec. 9 because these tests, according to our analysis, are diagnostic of beam offset. Our simulations based on the above offset values suggest a length error of about 250 μm, when a 2 m length is measured with the tracker about 0.5 m away from the reference length for both the horizontal and vertical orientations. With the tracker at the mirror position, we predict an error of equal magnitude but opposite sign. Our experimental values were 350 μm and −250 μm for the horizontal length tests, and 320 μm and −280 μm for the vertical length tests. These values are comparable to the simulation results; the slight discrepancy stems from other sources of misalignments that may also contribute to these tests. But overall, the errors are much larger than any of the errors shown in Fig. 9(a) and confirm the value of asymmetrical tests in tracker testing.

We note the following two points from Fig. 9. First, an offset in the beam along the *OM* axis should generally be sensitive to the length measurement system test at the user-defined default position 1, if the length is positioned as close as possible to the tracker. The measured error (see point # 34 in Fig. 9(a)) at this position is approximately 50 μm and within the MPE because the user-defined position 1 was performed at a non-optimal position of about 3 m from the tracker. This suggests a need for emphasizing the placement position of the reference length in default position 1 in the Standard.

Second, in Fig. 9(c), we note that the vertical angle plot shows a saw-tooth pattern indicating dependence on the azimuthal angle. This pattern, when scaled by the range, does indeed map on to the saw-tooth pattern in Fig. 9(b). While such dependence of two-face error on azimuth might be indicative of horizontal angle encoder eccentricity, the absence of a similar behavior in the length test results, particularly the horizontal length tests, suggests some other error source not modeled by the geometric error model might be the reason for the observed systematic error (for instance, internal stressing and relaxation of components such as cables may conceivably cause such observed behavior).

## 11. Conclusions

We have, in this paper, explored the relationship between geometric misalignments in the construction of laser trackers and performance evaluation tests described in the B89.4.19 Standard. It is desirable that tests described in any performance evaluation Standard be sensitive to all sources of systematic error including geometric misalignments. Our approach to the evaluation of the B89.4.19 tests uses geometric error models and numerical simulation. This requires the development of different error models for different mechanical designs, but that effort provides a systematic yet relatively straightforward numerical approach to understanding sensitivities.

Summarized here are some of our primary observations:

- Two-face system tests described in the Standard offer quick and fairly extensive diagnostic capability. There is considerable diagnostic value in recording actual errors in the angles in two-face system tests instead of convolving these errors into a distance error.
- Length measurement system tests described in the Standard are sensitive to numerous geometric misalignment parameters, but for certain tracker configurations, there may be room for improvement in the placement of the reference length artifact. We have proposed some new placement scenarios that improve sensitivity for certain tracker constructions and these tests may be considered as additional or optional tests.
- Two of the tests we have proposed already exist in some form in the Standard. These are the user-defined default position 1 and the horizontal length test in the near position. The verbiage in the Standard allows for flexibility in the placement of the reference lengths; non-optimal placement will reduce sensitivity to some geometric misalignment parameters as we demonstrated in Sec. 10.
- From a geometrical misalignment perspective, there are some redundant tests in the Standard, but we do realize that other systematic sources exist, and these tests may prove valuable. Modeling other systematic sources of error and additional testing is required prior to any recommendations for removing redundant tests.

As a final note, we realize that a number of interesting possibilities emerge as a result of the analysis methods described in this paper. Suitable artifact locations may be identified using sensitivity analysis for the determination of geometric misalignment parameters by best-fitting. Monte Carlo simulation in combination with error models may be used in the determination of the component of uncertainty in a length measurement from uncertainty in the error model parameters themselves. New international Standards development activity conceivably may benefit from such sensitivity analysis in optimal placement of artifacts within the work volume.

## Figures and Tables

**Table 1:** Sensitivity matrix for length measurement system tests (shaded boxes show non-zero sensitivity)^a^

				X1t	X1m	X2	X3	X4t	X5	X6x	X6y	X7n	X7z	X8	X9a	X9b	X10a	X10b
HOR	1 m	0	1	0.0	0.0	−1.5	0.0	0.0	0.0	0.0	1.5	0.0	0.0	−1.5	−2.0	0.0	0.0	0.0
	3 m	0	2	0.0	0.0	−0.7	0.0	0.0	0.0	0.0	2.1	0.0	0.0	−0.7	−4.0	0.0	0.0	0.0
		90	3	0.0	0.0	−0.7	0.0	0.0	0.0	2.1	0.0	0.0	0.0	−0.7	4.0	0.0	0.0	0.0
		180	4	0.0	0.0	−0.7	0.0	0.0	0.0	0.0	−2.1	0.0	0.0	−0.7	−4.0	0.0	0.0	0.0
		270	5	0.0	0.0	−0.7	0.0	0.0	0.0	−2.1	0.0	0.0	0.0	−0.7	4.0	0.0	0.0	0.0
	6 m	0	6	0.0	0.0	−0.4	0.0	0.0	0.0	0.0	2.3	0.0	0.0	−0.4	−4.4	0.0	0.0	0.0
		90	7	0.0	0.0	−0.4	0.0	0.0	0.0	2.3	0.0	0.0	0.0	−0.4	4.4	0.0	0.0	0.0
		180	8	0.0	0.0	−0.4	0.0	0.0	0.0	0.0	−2.3	0.0	0.0	−0.4	−4.4	0.0	0.0	0.0
		270	9	0.0	0.0	−0.4	0.0	0.0	0.0	−2.3	0.0	0.0	0.0	−0.4	4.4	0.0	0.0	0.0

VER	3 m	0	10	0.0	0.0	0.0	0.0	0.0	0.0	0.0	0.0	2.1	0.0	−0.7	0.0	0.0	4.0	0.0
		90	11	0.0	0.0	0.0	0.0	0.0	0.0	0.0	0.0	2.1	0.0	−0.7	0.0	0.0	4.0	0.0
		180	12	0.0	0.0	0.0	0.0	0.0	0.0	0.0	0.0	2.1	0.0	−0.7	0.0	0.0	4.0	0.0
		270	13	0.0	0.0	0.0	0.0	0.0	0.0	0.0	0.0	2.1	0.0	−0.7	0.0	0.0	4.0	0.0
	6 m	0	14	0.0	0.0	0.0	0.0	0.0	0.0	0.0	0.0	2.3	0.0	−0.4	0.0	0.0	4.4	0.0
		90	15	0.0	0.0	0.0	0.0	0.0	0.0	0.0	0.0	2.3	0.0	−0.4	0.0	0.0	4.4	0.0
		180	16	0.0	0.0	0.0	0.0	0.0	0.0	0.0	0.0	2.3	0.0	−0.4	0.0	0.0	4.4	0.0
		270	17	0.0	0.0	0.0	0.0	0.0	0.0	0.0	0.0	2.3	0.0	−0.4	0.0	0.0	4.4	0.0

RD	3 m	0	18	0.0	0.0	−0.4	0.0	0.0	−1.1	0.0	1.1	1.0	0.0	−0.7	−2.1	0.0	2.0	0.0
		90	19	0.0	0.0	−0.4	0.0	0.0	−1.1	1.1	0.0	1.0	0.0	−0.7	2.1	0.0	2.0	0.0
		180	20	0.0	0.0	−0.4	0.0	0.0	−1.1	0.0	−1.1	1.0	0.0	−0.7	−2.1	0.0	2.0	0.0
		270	21	0.0	0.0	−0.4	0.0	0.0	−1.1	−1.1	0.0	1.0	0.0	−0.7	2.1	0.0	2.0	0.0
	6 m	0	22	0.0	0.0	−0.2	0.0	0.0	−1.1	0.0	1.1	1.1	0.0	−0.4	−2.3	0.0	2.2	0.0
		90	23	0.0	0.0	−0.2	0.0	0.0	−1.1	1.1	0.0	1.1	0.0	−0.4	2.3	0.0	2.2	0.0
		180	24	0.0	0.0	−0.2	0.0	0.0	−1.1	0.0	−1.1	1.1	0.0	−0.4	−2.3	0.0	2.2	0.0
		270	25	0.0	0.0	−0.2	0.0	0.0	−1.1	−1.1	0.0	1.1	0.0	−0.4	2.3	0.0	2.2	0.0

LD	3 m	0	26	0.0	0.0	−0.4	0.0	0.0	1.1	0.0	1.1	1.0	0.0	−0.7	−2.1	0.0	2.0	0.0
		90	27	0.0	0.0	−0.4	0.0	0.0	1.1	1.1	0.0	1.0	0.0	−0.7	2.1	0.0	2.0	0.0
		180	28	0.0	0.0	−0.4	0.0	0.0	1.1	0.0	−1.1	1.0	0.0	−0.7	−2.1	0.0	2.0	0.0
		270	29	0.0	0.0	−0.4	0.0	0.0	1.1	−1.1	0.0	1.0	0.0	−0.7	2.1	0.0	2.0	0.0
	6 m	0	30	0.0	0.0	−0.2	0.0	0.0	1.1	0.0	1.1	1.1	0.0	−0.4	−2.3	0.0	2.2	0.0
		90	31	0.0	0.0	−0.2	0.0	0.0	1.1	1.1	0.0	1.1	0.0	−0.4	2.3	0.0	2.2	0.0
		180	32	0.0	0.0	−0.2	0.0	0.0	1.1	0.0	−1.1	1.1	0.0	−0.4	−2.3	0.0	2.2	0.0
		270	33	0.0	0.0	−0.2	0.0	0.0	1.1	−1.1	0.0	1.1	0.0	−0.4	2.3	0.0	2.2	0.0

UD1			34	0.0	0.6	0.0	0.0	0.0	0.0	0.0	0.0	0.9	0.6	−0.9	0.0	0.0	0.7	1.7
UD2			35	−0.1	−0.2	−0.1	0.0	0.0	−0.8	0.0	0.6	1.1	0.0	−0.3	−1.1	0.0	2.2	0.1

			A1	0.8	0.0	−1.0	0.0	0.0	0.0	0.4	0.5	0.0	0.0	−1.0	−0.2	1.0	0.0	0.0
			A2	−0.8	0.0	−1.0	0.0	0.0	0.0	−0.4	0.5	0.0	0.0	−1.0	−0.2	−1.0	0.0	0.0
			B1	0.0	0.6	0.0	0.0	0.0	0.0	0.0	0.0	0.9	0.6	−0.9	0.0	0.0	0.7	1.7
			B2	0.0	−0.6	0.0	0.0	0.0	0.0	0.0	0.0	0.9	−0.6	−0.9	0.0	0.0	0.7	−1.7
			C1	0.5	0.5	−0.7	−0.8	0.8	1.2	0.2	0.3	0.3	1.1	−1.4	−0.2	0.6	0.2	1.5
			C2	−0.5	−0.5	−0.7	0.8	−0.8	1.2	−0.2	0.3	0.3	−1.1	−1.4	−0.2	−0.6	0.2	−1.5
			D1	0.0	1.3	−2.0	−2.0	0.0	0.0	0.0	0.0	−1.3	1.5	−1.5	0.0	0.0	−2.0	0.3
			D2	0.0	1.1	−1.8	−1.8	0.0	0.0	0.0	0.9	−1.1	1.4	−1.4	−0.7	0.0	−1.8	0.4
			E	0.0	0.0	−2.0	0.0	0.0	0.0	0.0	0.0	0.0	0.0	−2.0	0.0	0.0	0.0	0.0

a The 35 length measurement system tests in Table 1 are in the order in which they appear in the Standard. Test 1 is the horizontal length test at the near position (1 m away, azimuthal angle of 0°). Tests 2 through 5 are the horizontal lengths at four orientations of the tracker (0°, 90°, 180° and 270°) at the 3 m distance. Tests 6 through 9 are the horizontal lengths at four orientations of the tracker at the 6 m distance. Tests 10 through 17 are the vertical length tests. Tests 18 through 25 are the right diagonal lengths and tests 26 through 33 are the left diagonal lengths. Tests 34 and 35 are the user-defined positions. The subsequent 9 rows marked as tests A1, A2, B1, B2, C1, C2, D1, D2 and E are not described in the Standard; these are tests we propose in Sec. 9.

**Table 2:** Sensitivity matrix for two-face system tests (shaded boxes show non-zero sensitivity)^a^

				X1t	X1m	X2	X3	X4t	X5	X6x	X6y	X7n	X7z	X8	X9a	X9b	X10a	X10b
1m	LOW	0	1	2.0	2.0	1.7	3.6	3.6	3.0	2.0	0.0	3.0	0.0	0.0	0.0	0.0	0.0	0.0
		90	2	2.0	2.0	1.7	3.6	3.6	3.0	0.0	2.0	3.0	0.0	0.0	0.0	0.0	0.0	0.0
		180	3	2.0	2.0	1.7	3.6	3.6	3.0	2.0	0.0	3.0	0.0	0.0	0.0	0.0	0.0	0.0
		270	4	2.0	2.0	1.7	3.6	3.6	3.0	0.0	2.0	3.0	0.0	0.0	0.0	0.0	0.0	0.0
	MED	0	5	2.0	2.0	0.0	2.0	2.0	0.0	2.0	0.0	0.0	0.0	0.0	0.0	0.0	0.0	0.0
		90	6	2.0	2.0	0.0	2.0	2.0	0.0	0.0	2.0	0.0	0.0	0.0	0.0	0.0	0.0	0.0
		180	7	2.0	2.0	0.0	2.0	2.0	0.0	2.0	0.0	0.0	0.0	0.0	0.0	0.0	0.0	0.0
		270	8	2.0	2.0	0.0	2.0	2.0	0.0	0.0	2.0	0.0	0.0	0.0	0.0	0.0	0.0	0.0
	HIGH	0	9	2.0	2.0	1.7	3.6	3.6	3.0	2.0	0.0	3.0	0.0	0.0	0.0	0.0	0.0	0.0
		90	10	2.0	2.0	1.7	3.6	3.6	3.0	0.0	2.0	3.0	0.0	0.0	0.0	0.0	0.0	0.0
		180	11	2.0	2.0	1.7	3.6	3.6	3.0	2.0	0.0	3.0	0.0	0.0	0.0	0.0	0.0	0.0
		270	12	2.0	2.0	1.7	3.6	3.6	3.0	0.0	2.0	3.0	0.0	0.0	0.0	0.0	0.0	0.0

3m	LOW	0	13	2.0	2.0	0.9	6.7	6.7	3.0	6.0	0.0	3.0	0.0	0.0	0.0	0.0	0.0	0.0
		90	14	2.0	2.0	0.9	6.7	6.7	3.0	0.0	6.0	3.0	0.0	0.0	0.0	0.0	0.0	0.0
		180	15	2.0	2.0	0.9	6.7	6.7	3.0	6.0	0.0	3.0	0.0	0.0	0.0	0.0	0.0	0.0
		270	16	2.0	2.0	0.9	6.7	6.7	3.0	0.0	6.0	3.0	0.0	0.0	0.0	0.0	0.0	0.0
	MED	0	17	2.0	2.0	0.0	6.0	6.0	0.0	6.0	0.0	0.0	0.0	0.0	0.0	0.0	0.0	0.0
		90	18	2.0	2.0	0.0	6.0	6.0	0.0	0.0	6.0	0.0	0.0	0.0	0.0	0.0	0.0	0.0
		180	19	2.0	2.0	0.0	6.0	6.0	0.0	6.0	0.0	0.0	0.0	0.0	0.0	0.0	0.0	0.0
		270	20	2.0	2.0	0.0	6.0	6.0	0.0	0.0	6.0	0.0	0.0	0.0	0.0	0.0	0.0	0.0
	HIGH	0	21	2.0	2.0	0.9	6.7	6.7	3.0	6.0	0.0	3.0	0.0	0.0	0.0	0.0	0.0	0.0
		90	22	2.0	2.0	0.9	6.7	6.7	3.0	0.0	6.0	3.0	0.0	0.0	0.0	0.0	0.0	0.0
		180	23	2.0	2.0	0.9	6.7	6.7	3.0	6.0	0.0	3.0	0.0	0.0	0.0	0.0	0.0	0.0
		270	24	2.0	2.0	0.9	6.7	6.7	3.0	0.0	6.0	3.0	0.0	0.0	0.0	0.0	0.0	0.0

6 m	LOW	0	25	2.0	2.0	0.5	12.4	12.4	3.0	12.0	0.0	3.0	0.0	0.0	0.0	0.0	0.0	0.0
		90	26	2.0	2.0	0.5	12.4	12.4	3.0	0.0	12.0	3.0	0.0	0.0	0.0	0.0	0.0	0.0
		180	27	2.0	2.0	0.5	12.4	12.4	3.0	12.0	0.0	3.0	0.0	0.0	0.0	0.0	0.0	0.0
		270	28	2.0	2.0	0.5	12.4	12.4	3.0	0.0	12.0	3.0	0.0	0.0	0.0	0.0	0.0	0.0
	MED	0	29	2.0	2.0	0.0	12.0	12.0	0.0	12.0	0.0	0.0	0.0	0.0	0.0	0.0	0.0	0.0
		90	30	2.0	2.0	0.0	12.0	12.0	0.0	0.0	12.0	0.0	0.0	0.0	0.0	0.0	0.0	0.0
		180	31	2.0	2.0	0.0	12.0	12.0	0.0	12.0	0.0	0.0	0.0	0.0	0.0	0.0	0.0	0.0
		270	32	2.0	2.0	0.0	12.0	12.0	0.0	0.0	12.0	0.0	0.0	0.0	0.0	0.0	0.0	0.0
	HIGH	0	33	2.0	2.0	0.5	12.4	12.4	3.0	12.0	0.0	3.0	0.0	0.0	0.0	0.0	0.0	0.0
		90	34	2.0	2.0	0.5	12.4	12.4	3.0	0.0	12.0	3.0	0.0	0.0	0.0	0.0	0.0	0.0
		180	35	2.0	2.0	0.5	12.4	12.4	3.0	12.0	0.0	3.0	0.0	0.0	0.0	0.0	0.0	0.0
		270	36	2.0	2.0	0.5	12.4	12.4	3.0	0.0	12.0	3.0	0.0	0.0	0.0	0.0	0.0	0.0

a The 36 two-face system tests in Table 2 are in the order in which they appear in the B89.4.19 Standard. Therefore, tests 1 through 4 are the two-face system tests at the near position (1 m) with the target on the floor for four orientations of the tracker (0°, 90°, 180° and 270°). Tests 5 through 8 are the two-face system tests at the near position (1 m) with the target at tracker height for four orientations of the tracker. Tests 9 through 12 are the two-face system tests at the near position (1 m) with the target at twice the tracker height for four orientations of the tracker (0°, 90°, 180° and 270°). Tests 13 through 24 are a repetition of tests 1 through 12 but with the tracker 3 m away from the target. Tests 25 through 36 are a repetition of tests 1 through 12 but with the tracker 6 m away from the target.
